# Development and validation of a predictive model for poor prognosis of communication disorders in children with cerebral palsy after cervical perivascular sympathectomy

**DOI:** 10.1007/s10143-024-02380-6

**Published:** 2024-04-08

**Authors:** Junjie Wu, Chao Bai, Baofeng Yan, Nurehemaiti Mutalifu, Qi Guan, Jianglong Li, Xinping Luan

**Affiliations:** Department of Cerebral Palsy Center in Neurosurgery, Second Affiliated Hospital of Xinjiang Medical University, Nanhu North Road, Shuimogou District, Urumqi, Xinjiang 830063 China

**Keywords:** Cerebral palsy, Cervical perivascular sympathectomy, Communication function, Prediction model, Prognosis

## Abstract

Cervical perivascular sympathectomy (CPVS) can improve communication disorders in children with cerebral palsy (CP); however, there are no research reports on the factors affecting surgical efficacy. This study aimed to establish a nomogram for poor prognosis after CPVS. We collected data from 313 CP patients who underwent CPVS at the Neurosurgery Cerebral Palsy Center of the Second Affiliated Hospital of Xinjiang Medical University from January 2019 to January 2023. Among them, 70% (*n* = 216) formed the training cohort and 30% (*n* = 97) the validation cohort. The general data and laboratory examination data of both groups were analyzed. In training cohort, 82 (37.96%) showed improved postoperative communication function. Logistic analysis identified motor function, serum alkaline phosphatase, serum albumin, and prothrombin activity as the prognostic factors. Using these four factors, a prediction model was constructed with an area under the curve (AUC) of 0.807 (95% confidence interval [CI], 0.743–0.870), indicating its ability to predict adverse outcomes after CPVS. The validation cohort results showed an AUC of 0.76 (95% CI, 0.650–0.869). The consistency curve and Hosmer–Lemeshow test (*χ*^*2*^ = 10.988 and *p* = 0.202, respectively) demonstrated good consistency between the model-predicted incidence and the actual incidence of poor prognosis. Motor function, serum alkaline phosphatase, serum albumin, and prothrombin activity are independent risk factors associated with the prognosis of communication disorders after CPVS. The combined prediction model has a good clinical prediction effect and has promising potential to be used for early prediction of prognosis of CPVS.

## Introduction

Cerebral palsy (CP) is not a distinct and separate disease classification but rather a collective term encompassing various symptoms and causes that vary with age [1]. It is the most common physical disability among children, and its prevalence among children with polio is approximately 0.15–0.36% [2]. The motor characteristics of CP include single or combined hypotonia, spasms, weakness, and involuntary movements, with motor disorders being the most common manifestation [3, 4]. In addition to the motor symptoms, CP can cause communication disorders due to potential issues in language, speech, or hearing [5]. Previous studies have indicated that 58–81% of CP patients with mild to moderate motor dysfunction (Gross Motor Function Classification System [GMFCS] Levels I–III) and 100% of CP patients with severe motor dysfunction (GMFCS Levels IV–V) experience communication disorders [6].

Communication disorders in children with CP create obstacles to their education and integration into society, leading to a substantial reduction in their overall quality of life [7]. Previous studies have demonstrated that the 6-month improvement rate of communication function in children with athetoid CP after CPVS is 45.6% [8], indicating that CPVS can enhance their communication functions. However, it is important to note that the surgical efficacy is only 45%, implying that not all CP patients benefit from surgery.

Reports have identified factors that influence CPVS in improving outcomes CP [9]. To the best of our knowledge, no published studies exist on the use of a nomogram for preoperative evaluation of communication disorder prognosis. Therefore, this study aimed to collect basic preoperative data and laboratory examination data of children with CP to construct a predictive model of the poor prognosis of communication disorders after CPVS and to provide preoperative prognostic information to clinical physicians. This will help to choose and develop more reasonable and effective treatment plans to improve communication function in children with CP.

## Materials and methods

### Sample sources

The study initially enrolled children who visited the Neurosurgery Cerebral Palsy Center of the Second Affiliated Hospital of Xinjiang Medical University between January 2019 and January 2023. The inclusion criteria for this study were as follows: (i) fulfillment of the internationally recognized CP diagnostic and staging criteria [10]; (ii) varying degrees of communication dysfunction; and (iii) availability of complete preoperative general information of the children with CP. The exclusion criteria were as follows: (i) having other neurological conditions, such as hydrocephalus, stroke, or meningitis, which may cause impaired communication; (ii) presence of other disorders in CP patients that affect communication; (iii) inability to undergo clinical examination and surgery under general anesthesia; and (iv) refusal for follow-up by the family; and (v) follow-up was less than one year. A total of 313 children were included in the study based on these criteria. Additionally, 70% of the participants were randomly assigned to the training group, while the remaining 30% were assigned to the validation group using the ‘randomizr’ function in R. The study was approved by the Medical Ethics Committee of the Second Affiliated Hospital of Xinjiang Medical University (Approval No. 2022H015), and informed consent was obtained from the patients and their family members for the surgical procedures and for all other treatment.

### Data collection and follow-up

We included the following variables by summarizing the available studies. We collected the following clinical data of children with CP: sex, age, intellectual disability, GMFCS level, drooling, epilepsy, history of neonatal asphyxia, gestational weeks, birth weight, serum alkaline phosphatase (ALP), serum albumin, serum iron, serum phosphorus, fibrinogen degradation products (FDP), activated partial thromboplastin time (APTT), thrombin time (TT), prothrombin time (PT), fibrinogen (FBG), D-dimer (DD), prothrombin activity (PTA), and blood type.

The study had a follow-up period ranging from 16 to 48 months, with a median follow-up of 26 months.

### Definitions

The Communication Function Classification System (CFCS) was used to characterize the communication ability of the participants [11]. The CFCS describes the communication abilities of children with CP in a reliable and valid manner [11]. The CFCS ranges from Level I to Level V, with Level I indicating “effective sending and receiving with unknown and known partners” and Level V indicating “rarely effective sending and receiving even with known partners”. The CFCS was used to assess the communication ability of children with CP preoperatively. After surgery, the children’s communication skills were re-evaluated using the CFCS. The children’s preoperative and postoperative communication abilities were compared. A decrease of ≥ 1 point in the CFCS level indicated a good prognosis, whereas no change or an increase in the CFCS level indicated a poor prognosis [9].

Motor function in children with CP has been characterized using the GMFCS [12]. The GMFCS consists of five levels, each with different scoring criteria based on age group. Level I represents the highest level of function, indicating independent walking in all settings, while level V represents the lowest level of function, indicating the need for a wheelchair for all mobility. In this study, we defined GMFCS levels I-III as mild to moderate and GMFCS levels IV-V as severe.

Intellectual disability is defined as a previous diagnosis of intellectual disability or IQ < 70 [13].

### Surgical methods

The surgical procedures were conducted by an experienced physician. Following intravenous general anaesthesia and tracheal intubation, the patient was positioned supine with shoulder pads and the head tilted towards one side. After regular disinfection, a 3.0 cm-long transverse incision was made 1 cm below the thyroid cartilage (medial margin of the lateral sternocleidomastoid muscle). Afterwards, an incision was made layer by layer into the skin, with the platysma being longitudinally separated subcutaneously. After cutting through the superficial layer of cervical fascia, the surgeon pulled the sternocleidomastoid muscle laterally and drew the thyrohyoid muscle medially. The carotid artery is exposed by incising the carotid sheath 6 cm below the carotid bifurcation. Care is taken to protect the jugular vein and vagus nerve. The outer layer of the common carotid artery is then separated on both sides, heading caudally then rostrally, until the carotid bifurcation is reached. The internal and external carotid arteries are then separated. The surgical dissection is done under microscopy to protect the surrounding tissue and the possibility of damage from excessive stretching. Thorough hemostasis is obtained following the dissection of the carotid artery. Finally, the closure of the incision is done in a multi-layer fashion.

### Statistical analysis

SPSS software version 25 (IBM, Chicago, USA) was utilized for statistical analysis. Mean ± standard deviation are used to present quantitative data, which were compared by t-test. Qualitative data are presented as relative numbers and compared using the *χ*^*2*^ test. Normality tests were conducted using the Kolmogorov-Smirnov test. Quantitative data, which did not follow a normal distribution, and rank data were analyzed using the rank-sum test. *P*-values less than 0.05 were deemed statistically significant. G*Power was used to calculate the study’s true power, which was 0.8031 with a sample size of 85 (parameters: effect size: 0.15, α err prob: 0.05, power: 0.8, number of predictors: 4).

The following steps were used to develop and validate a nomogram for predicting the adverse outcomes after CPVS. First, univariate analysis was conducted to test potential predictive factors. The factors with a *p*-value < 0.2 in the univariate analysis were included in the multivariate analysis. Second, risk factors for poor communication disorder outcomes were gradually included through multivariate analysis. For the continuous variables, the cut-off value was determined using the receiver operating characteristic (ROC) curve and adjusted to the second categorical variable. The risk factors were incorporated, and a logistic regression model was established using the outcome of communication disorders as a predictive indicator. Finally, based on the selected risk factors through binary logistic regression testing, a nomogram was constructed. The accuracy of nomogram prediction was evaluated using the ROC, concordance index, calibration curves, and decision curves (DCA) analysis.

## Results

### Efficacy of CPVS

Among the 216 children with CP who underwent CPVS, 134 had a poor prognosis for their communication disorder, while 82 had a good prognosis (37.96%).

### Univariate analysis of factors influencing surgical efficacy

The good prognosis group and poor prognosis group showed no difference in age, sex, epilepsy, salivation, neonatal asphyxia, premature birth, low birth weight, serum iron, serum phosphorus, FDP, APTT, TT, PT, FBG, DD, or blood type. However, they showed statistically significant differences in motor dysfunction, intellectual disability, serum albumin, serum ALP, and PTA (Table 1).

**Table 1:** Univariate analysis of factors influencing surgical efficacy

Factors (self-variable name)	Good prognosis (*n* = 82)	Poor prognosis (*n* = 134)	χ^2^/Z/t	p-Value
Age(Years)	7(4,9)	6(4,8.25)	0.86	0.38
*Gender*				
Male (= 0)	49	77		
Female (= 1)	33	57	0.74	0.43
*Intellectual disability*				
Yes(=0)	66	123		
No(=1)	16	11	5.94	0.01
*Epilepsy*				
No (= 0)	61	97		
Yes (= 1)	21	37	0.10	0.74
*Drooling*				
No (= 0)	30	56		
Yes (= 1)	52	78	0.57	0.44
*GMFCS*				
Level I-III (= 0)	55	51		
Level IV-V (= 1)	27	83	17.13	<0.001
*Neonatal asphyxia*				
No (= 0)	43	76		
Yes (= 1)	39	59	0.31	0.580
*Preterm birth*				
Yes (= 0)	24	31		
No (= 1)	58	103	1.008	0.315
*Birth weight (gram)*				
<2500	23	38		
≥ 2500	59	96	0.002	0.961
BMI	16.6(14.96,17.96)	16.3(14.5,18.3)	0.749	0.454
Serum iron (umol/L)	11.5(8.3,16.3)	12.2(8.2,15.4)	0.925	0.768
Serum Phosphorus (umol/L)	1.86(1.74,2.02)	1.87(1.70,2.04)	0.258	0.796
Serum alkaline phosphatase (u/L)	223(202,249)	149(129,171)	12.35	<0.001
Serum albumin (g/l)	43.4(40.4,46.1)	41.5(39.1,44.2)	2.901	0.004
FDP (ug/L)	2.05(1.10,2.79)	1.9(1.14,2.79)	0.88	0.930
APTT (second)	32.35(29.6,35.07)	33.1(29.79,36.35)	0.824	0.410
TT (second)	17(16.05,18.40)	17(16,18.15)	1.031	0.303
PT (second)	12.9(12,13.6)	12.9(12.1,13.4)	0.404	0.686
Fbg (g/L)	2.69(2.35,3.1)	2.6(2.21,2.99)	1.117	0.264
DD (mg/L)	0.3(0.19,0.55)	0.31(0.19,0.51)	0.145	0.885
PTA (%)	99(84.5,105)	96(87.75,106)	2.202	0.028
*Blood type*				
O	31	39		
Non O	51	95	1.758	0.185

### Multivariate analysis of factors influencing surgical efficacy

In the multivariate analysis, the following factors were included: intellectual disability, motor dysfunction, serum albumin, serum ALP, blood type, and PTA. The cut-off values for serum albumin, serum ALP, and PTA were 43.55 g/L, 194 u/L, and 97.6%, respectively. Multivariate analysis revealed that motor dysfunction, serum ALP, serum albumin, and PTA were the influential factors for poor prognosis of communication disorders in children with CP after CPVS (Table 2).

**Table 2:** Logistic regression analysis for multiple factors affecting the efficacy of CPVS

					Exp (B) 95% C.I.	
Factors	*B* value	S.E. value	*p*-Value	Exp (*B*)	Lower limit	Upper limit
GMFCS	-0.888	0.345	0.010	0.412	0.209	0.810
Intellectual disability	-0.736	0.502	0.142	0.479	0.179	1.280
Serum alkaline phosphatase	1.002	0.342	0.003	2.724	1.394	5.322
Serum albumin	1.609	0.348	0.000	4.997	2.526	9.883
Prothrombin Activity	1.008	0.346	0.002	2.696	1.506	5.853
Blood type	-0.714	0.367	0.052	0.490	0.238	1.005

### Development, validation, and assessment of the nomogram

**Fig. 1:**
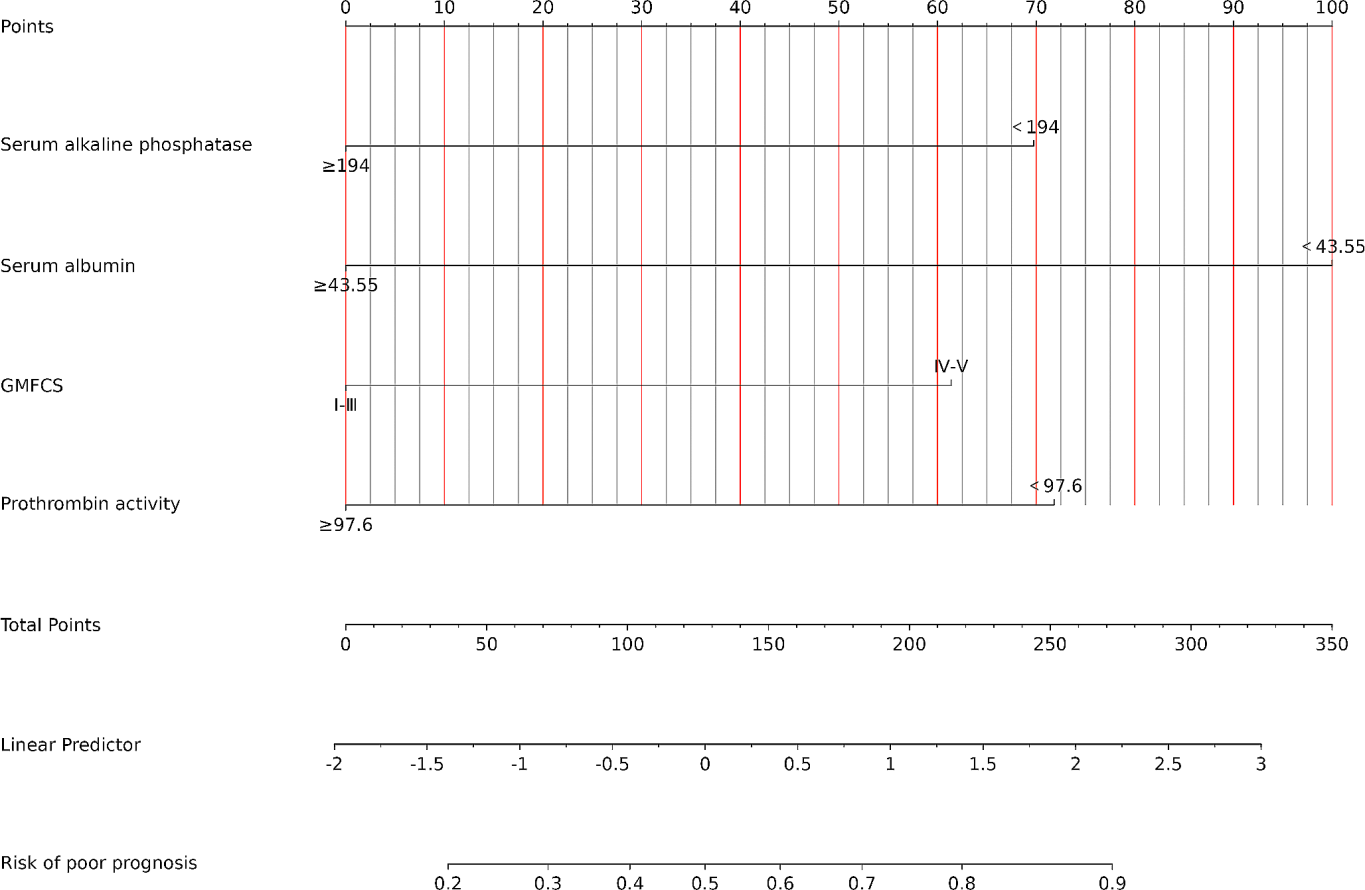
Nomogram to predict the probability of poor communication outcomes in CP after CPVS. A nomogram was constructed using four factors from the training cohort, including GMFCS level, serum albumin (<43.55 g/l), serum alkaline phosphatase (<194u/L), and prothrombin activity (<97.6%). The total score is obtained by adding the corresponding point scores of the four indicators, and the probability corresponding to the total score is found, which is the probability of the outcome of poor communication function after CPVS

**Fig. 2:**
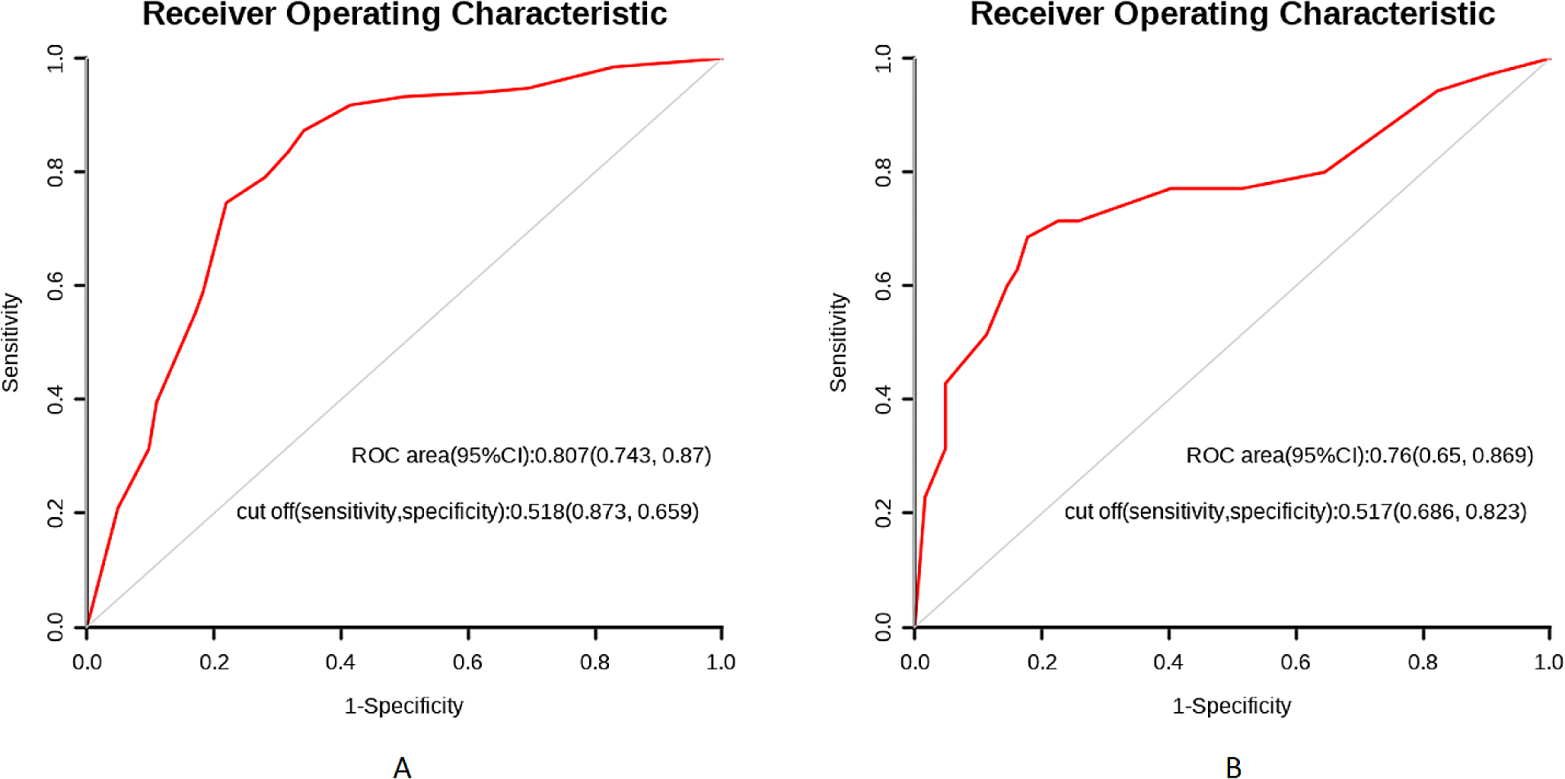
(**A**) In the training cohort, the AUC of the nomogram used to predict the outcome of poor communication function in children with CP after CPVS, was 0.807(95%CI: 0.734–0.870). (**B**) In the validation cohort, the AUC of the nomogram, used to predict the outcome of poor communication function in children with CP after CPVS, was 0.760(95%CI:0.650–0.869)

**Fig. 3:**
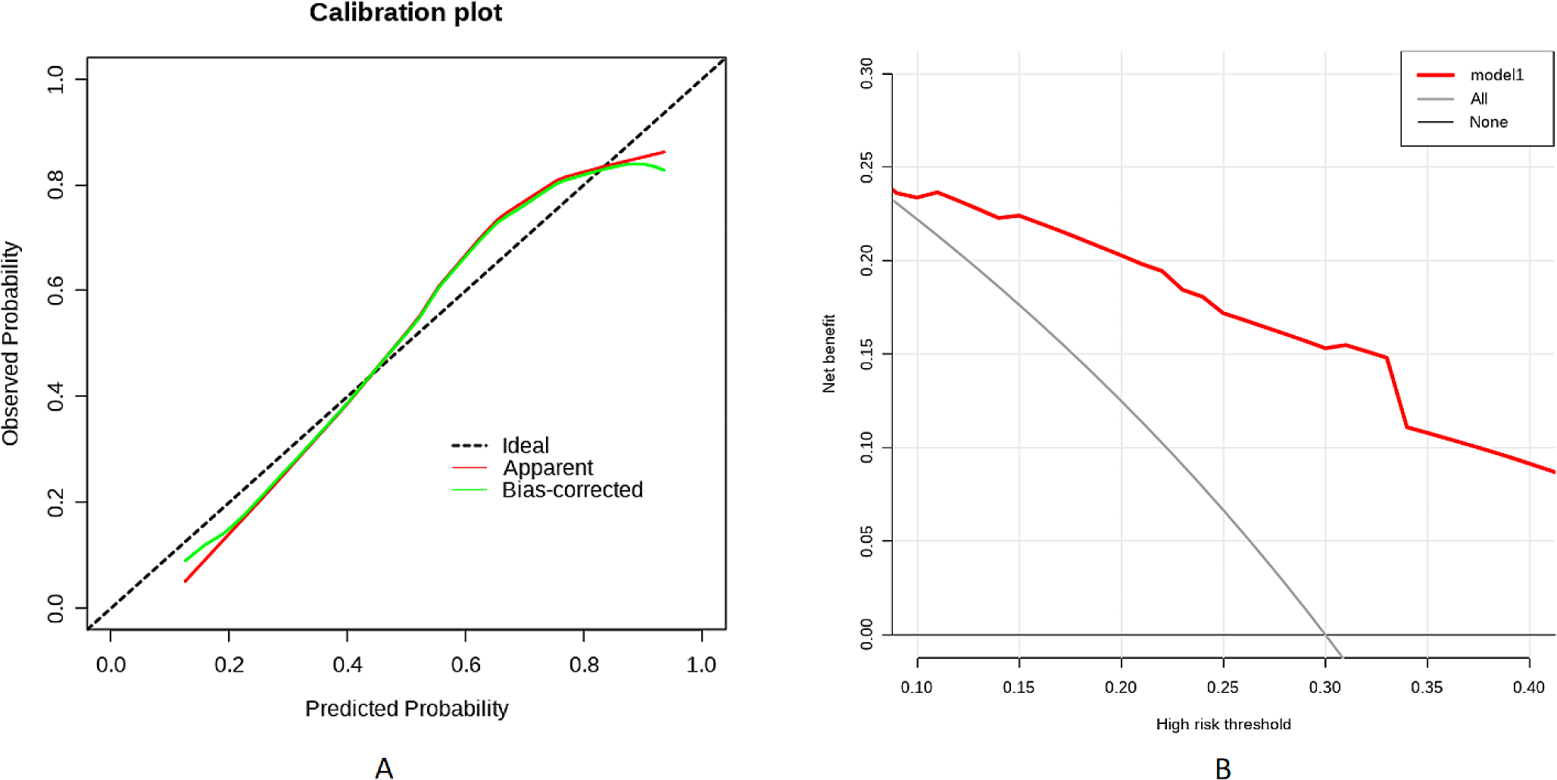
(**A**) Calibration curve and the Hosmer-Lemeshow test (*χ*^*2*^ = 10.988, *p* = 0.202) showed that the model predicted and the actual incidence rate were in good agreement. (**B**) The DCA was utilized to assess the predictive nomogram’s performance across various threshold probabilities. Net benefits were measured and compared. The predictive nomogram is depicted by the red line, while the gray line represents the scenario where all patients experience unsuccessful treatment outcomes. Conversely, the black line represents the assumption that no patients encounter unsuccessful treatment outcomes

Based on the results of the multivariate regression analysis, a nomogram was constructed using the four influential factors (Fig. 1). The areas under the ROC curve (AUC) and the calibration curve were used to evaluate the model. The AUC for predicting the outcome of communication disorder after CPVS was 0.807 (95% confidence interval [CI], 0.743–0.87) (Fig. 2A), with a sensitivity of 0.873 and specificity of 0.659 when the Jordon index was 0.518. The AUC of the validation cohort was 0.76 (95% CI, 0.65–0.869) (Fig. 2B), with a sensitivity of 0.823 and specificity of 0.686 when the Jordon index was 0.517. We plotted a calibration curve (Fig. 3A) to assess the accuracy of the model, and the calibration curve and the Hosmer-Lemeshow test (*χ*^*2*^ = 10.988 and *p* = 0.202, respectively) showed that the model-predicted incidence and actual incidence of poor prognosis were in good agreement. Finally, the DCA (Fig. 3B) suggested that patients could benefit from the model when the threshold probability is 0.04–0.64.

### Postoperative complications of CPVS

Following surgery, eight patients experienced subcutaneous or intramuscular hematoma (2.5%) which resolved within 2 to 7 days post-surgery. Additionally, 25 patients (7.9%) showed fever that disappeared three days after surgery. Mild skin burns were observed in six patients (1.9%), while one patient (0.31%) developed a persistent cough related to drinking.

## Discussion

CP is a prevalent neurological disorder among children that is typified by postural abnormalities, central motor dysfunction, and communication disorders commonly present. Studies have indicated that a significant proportion of children with CP, ranging from 33 to 66%, experience communication dysfunction [14]. Previous research has reported a 6-month improvement rate of 45% in the communication function of CP patients after CPVS [8]. However, in this study, the recovery rate of the communication function was slightly low at 37.96%. This discrepancy between the studies could be attributed to the different assessment methods used. This study employed a scale to evaluate postoperative recovery, whereas previous studies relied on parental evaluation of surgical success [8].

Both studies highlight the fact that more than half of the patients with communication disorders did not experience improvement after surgery. Therefore, it is crucial to predict the prognosis of patients before surgery to avoid subjecting them and their families to unnecessary burdens when the potential benefits are limited. This study utilized general patient information and selected laboratory test results. After a gradual screening process, a nomogram was constructed for communication disorder outcomes in children with CP after CPVS. The aim of developing this predictive model is to provide clinicians with a tool to assess the potential outcomes of CPVS in terms of communication function. By identifying patients who are less likely to benefit from the surgery, appropriate counseling and alternative intervention strategies can be offered to optimize patient care and reduce the burden on families.

This study revealed that severe motor dysfunction (GMFCS Levels IV–V) in children with CP is an independent predictor of poor prognosis of communication disorders after CPVS. Previous research demonstrated that children with CP classified as GMFCS Level V have significant motor impairments along with the inability to resist gravity, inability to engage in autonomous movement, and a complete loss of motor function [15]. This indicates that these patients have severe brain damage, limited functional plasticity in neuroplasticity, and a low likelihood of recovery of communication function. Furthermore, our analysis of the original data indicated that most of these patients had limited or no speech ability before surgery. This finding aligns with the research conducted by Malandraki et al. [16] observed that children with unilateral spastic CP are more likely to experience dysphagia and motor aphasia than those showing typical development. These findings are consistent with the clinical data obtained in our study, suggesting that children with severe motor impairments in CP, particularly during school age, tend to exhibit severe communication disorders. Brain damage in these cases may involve nuclei related to communication centers, such as white and gray matter [17], leading to a poor prognosis for communication disorders. These results emphasize the importance of considering the severity of motor dysfunction and its potential impact on communication disorders in children with CP. By recognizing the potential limitations in functional plasticity and the involvement of communication-related brain regions, clinicians can manage the expectations better and develop targeted interventions to support communication development in these patients.

ALP comprises zinc (II) metalloenzymes that catalyze the hydrolysis of phosphate esters by forming phosphorylated serine intermediates [18]. Wang et al. [19] conducted a proteomic analysis to compare the serums of children with spastic CP and healthy controls and identified differentially expressed proteins. The gene expression product of tissue-nonspecific ALP, known as the tissue-nonspecific isozyme of ALP (TNAP), was downregulated in children with spastic CP. This decrease in the TNAP levels was also observed in a subsequent rat model of CP. TNAP acts as an inhibitor of nuclear factor kappa B (NF-κB) activity, and reduced TNAP expression can induce activation of the NF-κB pathway. Neuroinflammation caused by cerebral ischemia and hypoxia can have an abnormal impact on the NF-κB pathway, leading to the expression of inflammatory factors. Cerebral ischemia and hypoxia can trigger the NF-κB/interleukin (IL)-6 pathway, resulting in rapid release of IL-6 [20]. In addition, IL-17 can activate downstream signaling pathways like NF-κB, leading to the expression of pro-inflammatory chemokines and cytokines [21]. Therefore, the decrease in serum ALP levels may contribute to exacerbated central nervous system inflammation in children with CP, thereby affecting their prognosis [22]. This finding is consistent with our findings, which identified low ALP levels as a risk factor for poor language function outcomes.

In this study involving children with CP, the serum albumin levels were significantly higher in the group with good prognosis than in the group with poor prognosis of communication disorders after CPVS. Using the ROC curve, the optimal cut-off value for serum albumin, corresponding to the maximum Youden index, was determined as 43.55 g/L. Based on this optimal cut-off value, the serum albumin values were categorized into two groups. After binary multivariate logistic regression analysis, serum albumin < 43.55 g/L were considered independent predictors of poor prognosis after CPVS in CP.

Oxidative stress is implicated in the pathogenesis of numerous nervous system diseases. Impaired or increased oxidative capacity contributes to degenerative diseases [23], and serum antioxidant levels are associated with neural function. Kulak et al. [24] demonstrated significantly lower levels of antioxidant enzymes in children with CP than in the control patients, but no difference was observed in the lipid peroxide levels between them. Aycek et al. [25] investigated and compared the oxidative and antioxidant status of CP patients aged 1–12 years and healthy control patients. The study involved 42 CP patients and 7 healthy individuals. Lipid peroxidation was significantly higher in the CP group than in the control group. Moreover, the levels of serum total antioxidant capacity, uric acid, and albumin were significantly lower in the CP group than in the control group. The CP group demonstrated an increase in oxidants and a decrease in antioxidants, resulting in an imbalance favoring oxidative stress in children with CP.

These findings suggest that although the serum albumin levels in children with CP may be within the normal range, a correlation exists between serum albumin levels and the antioxidant capacity of children with CP. A decrease in serum albumin levels in these patients leads to a decline in the brain’s antioxidant capacity, exacerbating neurological damage and reducing the potential for brain function recovery. Therefore, in addition to removing damaging factors, children with CP who have experienced brain damage during the perinatal period should receive serum albumin supplementation and be treated with drugs that eliminate free radicals to enhance their antioxidant abilities and prevent subsequent nerve damage [26].

PTA is an important indicator of liver coagulation function and the degree of liver reserve function, which is closely related to the severity of liver disease [27]. The reduction of prothrombin activity often indicates the reduction or loss of coagulation factors, especially exogenous coagulation factors, or the use of anticoagulants by patients, such as heparin or warfarin, which affects the activity of the coagulation factors [28]. As for the increase in prothrombin activity, it may reflect a hypercoagulable state in the body, which can easily lead to thrombosis [29]. A Spanish study showed that in the coronavirus disease cohort, PTA was 23% higher in the good prognosis group than in the poor prognosis group [30]. Similarly, this study showed that low PTA is a risk factor for adverse prognostic outcomes. The relationship between coagulation and inflammation has been reported, and inflammation plays an important role in brain damage in children with CP [31]. Therefore, the role of coagulation factors in inflammation may affect the prognosis.

This study utilized a cohort study design to categorize children with CP undergoing CPVS into two groups based on the prognosis. It incorporated four clinically accessible indicators to develop a predictive model for poor prognosis of communication disorders following CPVS. As an example, a patient with significant motor dysfunction, a serum albumin level of 46 g/L, a PTA of 97%, and an alkaline phosphatase level of 227 u/L has a total score of 160 based on their condition. This score corresponds to a 53% probability of poor prognosis for their communication function after CPVS.

In clinical practice, CPVS may be able to be one of the therapeutic approaches to improve communication function in children with CP. For children with CP who have severe motor function impairment, a PTA of less than 97.6%, alkaline phosphatase levels of less than 194u/L, and serum albumin levels of less than 43.55 g/L, a more conservative approach to CPVS should be adopted. Such patients may benefit from comprehensive rehabilitation [32].

However, it is important to acknowledge several limitations of this study. First, previous research has demonstrated that CPVS can improve various functions, such as communication, salivation, and swallowing, in children with CP. This study focused solely on communication as the outcome measure; thus, future studies should consider including additional outcome factors. This study’s findings may not be generalizable due to the limited data from a single hospital. In this study, serum albumin and PTA were significant, but their clinical significance was questionable because they were not externally validated. So external validation of the model in other healthcare settings is necessary to enhance its robustness and applicability. Third, the sample size in this study was relatively small, which may introduce bias and affect the reliability of the results. To overcome this limitation, future studies including large-sized and more diverse cohorts are warranted to ensure that the findings are more accurate and representative. Thus, although this study presents a nomogram model for predicting the poor prognosis of communication disorders after CPVS in children with CP, it is important to address these limitations. Further research should expand the scope of outcome measures, validate the model in multiple centers, and increase the sample size to strengthen the current nomogram.

## Conclusion

This study established a predictive model for the recovery of communication function in children with CP after CPVS. The research results show that the model has good discrimination and calibration, which may provide crucial information for the recovery of communication function in CP after CPVS.

## Data Availability

Not applicable.
